# Maternal ethnicity, severe perinatal mental illness and involuntary admission: mother and baby unit service evaluation

**DOI:** 10.1192/bjb.2025.10182

**Published:** 2026-08

**Authors:** Katie F. M. Marwick, Zia J. Low, Kelly Fleetwood

**Affiliations:** 1 University of Edinburgh, Edinburgh, UK; 2 Perinatal Mental Health Service, NHS Lothian, Edinburgh, UK; 3 Acute Medicine, Raigmore Hospital, Inverness, UK; 4 Usher Institute, University of Edinburgh, Edinburgh, UK

**Keywords:** In-patient treatment, mental health services, perinatal psychiatry, psychotic disorders/schizophrenia, transcultural psychiatry

## Abstract

**Aims and method:**

To investigate associations between maternal ethnicity and involuntary mother and baby unit (MBU) admission, adjusting for potential confounding variables. Data from electronic records in a Scottish MBU (July 2012 to January 2024) were analysed with logistic regression.

**Results:**

A total of 450 first admissions were analysed. The proportion of patients from Black, Asian, Mixed or other ethnic minorities who were admitted involuntarily (*n* = 8/48, 38%) was twice that of White British patients (*n* = 66/364, 18%) with White not British patients showing an intermediate proportion (*n* = 12/38, 32%). In the unadjusted model, being of Black, Asian, Mixed or other minority ethnicity was associated with involuntary admission (odds ratio 2.7, 95% CI 1.4–5.2; *P* = 0.002), as was being of White not British ethnicity (odds ratio 2.1, 95% CI 1.0–4.3; *P* = 0.04997). Association were attenuated after adjustment for potential confounders, including psychosis.

**Clinical implications:**

We identified racial inequalities in a perinatal mental health setting. The drivers of these differences are likely multifactorial.

Perinatal mental disorders are the most common complication of pregnancy and are associated with long-term consequences for mothers and infants.^1^ In the UK, the most severe episodes of illness, where there is significant risk to the mother or child, are managed by admission to hospital. If the mother (or birthing person), is able to be the primary caregiver for their child, the preferred option is joint admission to a mother and baby unit (MBU).

Ethnicity is known to be associated with poorer maternal outcomes in the UK, with Black mothers having a nearly three-fold increased risk of maternal mortality compared with White mothers, and Asian mothers having a two-fold increase.^2^ Minority ethnicity mothers also have an increased risk of experiencing a common perinatal mental health condition,^3^ are less likely to have contact with perinatal community services^4^ and are more likely to be admitted to hospital involuntarily in England.^4^

This service evaluation therefore aimed to identify associations between maternal ethnicity or diagnosis and involuntary MBU admission. We also collected data on other factors of potential relevance to involuntary admission, and accounted for these statistically.

## Method

### Setting

Scotland is a devolved nation within the UK. It is a high-income country with a well-developed healthcare system, and has two MBUs. The MBU in Livingston is a six-bed ward that serves the population resident mainly in South-East Scotland: the Health Boards of Lothian, Fife, the Scottish Borders, Tayside and Highland. The majority of women admitted come from these areas, but the unit also accepts admissions from other health boards (Forth Valley and Grampian) and from the areas served by the West of Scotland MBU if this unit is full or a patient has a strong preference. The population served is largely of White ethnicity, with the 2011 census reporting that the total population of the regions primarily served by the unit was 1 955 746 people (rising to 2 128 543 in 2022), with average percentages (weighted by population size) of people self-identifying as White British being 91.1% (reducing to 86.0% in 2022), White not British being 5.2% (increasing to 7.3% in 2022) and other ethnicities being 3.8% (increasing to 6.7% in 2022).^5,6^ Data on maternal ethnicity by health board is publicly available from March 2020 to March 2024, showing a gradual increase in the proportion of mothers from Black, Asian, Mixed or other ethnicities from 8% in 2021 to 13% in 2024 (averaging at 9.8% of births over the 4 years) (in the 84% of births where ethnicity known).^7^

### Data source

Data were gathered from electronic records of admissions to the MBU in Livingston from its opening in July 2012 until January 2024. Data were collected on patients’ age, sex/gender (not distinguished between in the records), diagnosis, legal status (voluntary throughout admission or involuntary at any point during admission), ethnicity and need for an interpreter. All of these variables were collected routinely and are available as structured fields within patients’ electronic health records.

In cases where data on key variables were missing from the electronic record database (diagnosis, ethnicity or need for an interpreter), free-text electronic records were manually reviewed by a psychiatrist with membership of the Royal College of Psychiatrists (K.F.M.M.), and where available values for these variables were included in the data-set used for analysis.

Ethical approval and participant consent was not required as this was a service evaluation: no non-routine data were collected, no individual-level data were presented and no intervention was performed.

### Ethnicity categorisation

The entry in a patient’s electronic healthcare record is the patient’s self-reported ethnicity, entered by staff. Detailed regional ethnicity information was available, but to protect patient anonymity, ethnicity was broadly categorised into ‘White British’ (including all patients with White Scottish, White English or White British ethnicity), ‘White not British’ (including White patients from other regions, e.g. Ireland, North America, Europe, Australasia) and ‘Black, Asian, Mixed or other ethnicity’ (all other ethnicities, including those from Asia, Africa, Arab countries, Black British and Asian British, Mixed or multiple ethnicity), using the terminology recommended by the UK Government.^8^

### Diagnosis categorisation

Diagnoses were ICD-10 discharge diagnoses provided by the doctor primarily responsible for the patient’s care.

Diagnoses were categorised into five overarching categories: (a) psychosis (including drug-induced, non-affective and affective): F(10–19).5 (i.e. any substance(s) coded as associated with a psychotic disorder), F20–F29, F30.2, F30.8, F31.2, F31.5, F32.3, F33.3, F53.1; (b) mood disorder (excluding mood disorder with psychotic component): F30 (excluding F30.2), F31 (excluding F31.2 and F31.5), F32 (excluding F32.3), F33 (excluding F33.3), F34, F38, F39; (c) personality disorder: F60–69; (d) anxiety disorder including adjustment reactions: F40–48 and (e) other: any other ICD-10 code.

### Involuntary admission categorisation

Involuntary admission is regulated in Scotland by the Mental Health (Care and Treatment) (Scotland) Act 2003. Any new detention (Emergency Detention, Short Term Detention or Compulsory Treatment Order) at any point during the admission was classed as an involuntary admission.

### Statistical analysis

Statistical analysis was performed in R (version 4.3.1) in Windows.^9^ Associations between involuntary admission and theoretical predictors were first tested at the univariate level by chi-squared tests of independence, with associations with *P* > 0.2 included in adjusted logistic regression models. To meet the assumption of logistic regression that all observations are independent, only first admissions were included, and any repeat admissions were excluded from the data-set. The outcome variable was whether the admission was voluntary or involuntary. Simple (ethnicity as the only predictor) and adjusted logistic regression models (including other predictors) were fitted, followed by a *post hoc* model including an interaction term between two predictors. To compare goodness of fit of the models, McFadden’s pseudo-*R* ^2^ was calculated as (1 – the log likelihood of the full model/log likelihood of the intercept only model) (ranges 0–1, higher values better fit). To preserve patient anonymity, table cells with counts <5 were reported as <5 or categories merged.

## Results

### Data availability

Data on 584 admissions for 490 patients were reviewed. Structured data entry into the electronic healthcare record fields was generally good, with data missing for a minority of patients with regard to ethnicity (*n* = 62/584, 10.6%) and diagnosis (*n* = 83/584, 14.2%). Data on age and involuntary admission were complete. After manual review of the free-text records of those with missing data and inclusion of data where available, the sample size with full data available for all planned analyses was 539 admissions. A total of 18.2% (89/490) of patients had multiple admissions. Only their first admission was included in the data-set, giving a final full sample size of 450 admissions from 450 patients.

### Descriptive statistics

The proportion of patients of White British ethnicity admitted to the MBU was 81%, with 8% being of White other ethnicity and 11% having a Black, Asian, Mixed or other minority ethnic background (Table 1). The proportion of patients of non-British ethnicity was higher than expected, based on general population census ethnicity for the regions served by the unit and from data on maternal ethnicity available for a subset of the time period studied. Admission duration was very similar between groups. Just under a quarter of patients who were not White British required the use of an interpreter. A diagnosis of a psychotic illness was more common in Black, Asian, Mixed or other minority ethnicity patients (50%) and in White not British patients (45%) than in White British patients (30%). Mood disorders were less common in patients of Black, Asian, Mixed or other minority ethnicity than in patients of other ethnicities, but the frequency of anxiety disorders was similar between ethnicities. The proportion of Black, Asian, Mixed or other minority ethnicity patients who were admitted involuntarily (38%) was around twice that of White British patients (18%), with White not British patients experiencing an intermediate frequency (32%). More than zero, but fewer than five, patients identified as male gender.

**Table 1:** Demographic and clinical characteristics of women admitted for the first time to the Mother and Baby Unit (2012–2024), Livingston, Scotland Table 1 long description.

Ethnicity	White British	White not British	Black, Asian, Mixed or other ethnicity
*n* (% of total)	364 (80.9)	38 (8.4)	48 (10.7)
Age in years, *n* (% within group)			
≤24	103 (28.3)	7 (18.4)	9 (18.8)
25–29	94 (25.8)	9 (23.7)	18 (37.5)
30–34	85 (23.4)	12 (31.6)	16 (33.3)
≥35	82 (22.5)	10 (26.3)	5 (10.4)
Admission length in days, mean (s.d.)	29.4 (33.8)	32.2 (23.0)	29.0 (34.7)
Interpreter required, *n* (% within group)	0	9 (23.7)	11 (22.9)
Diagnosis, *n* (% within group)			
Psychotic disorder	109 (29.9)	17 (44.7)	24 (50.0)
Mood disorder	99 (27.2)	11 (28.9)	9 (18.8)
Anxiety disorder	62 (17.0)	5 (13.2)	8 (16.7)
Personality disorder, other or no disorder	94 (25.8)	5 (13.2)	7 (14.6)
Admitted involuntarily, *n* (%)	66 (18.1)	12 (31.6)	18 (37.5)

### Association between ethnicity and involuntary admission

Univariate analysis of theoretical predictors of involuntary admission showed a significant effect of ethnicity (*χ* ^2^(2) = 12.1, *P* = 0.0024), need for an interpreter (*χ* ^2^(1) = 8.5, *P* = 0.0034) and diagnosis of psychosis (*χ* ^2^(1) = 79.4, *P* < 0.001), but not of age (*χ* ^2^(3) = 1.2, *P* = 0.75). Of those who needed an interpreter, 50% (*n* = 10/20) were admitted involuntarily. Of those with a diagnosis of psychosis, 46% (*n* = 69/150) were admitted involuntarily. Ethnicity, need for an interpreter and diagnosis of psychosis were therefore analysed further with logistic regression.

A logistic regression (Table 2) showed that having a Black, Asian, Mixed or other ethnic minority background was significantly associated with involuntary admission, with an odds ratio of 2.7 (95% CI 1.4–5.2) relative to White British patients. The odds ratio for White not British patients was 2.1 (95% CI 1.0–4.3), which was also statistically significantly greater than that for White British patients. These odds ratios were attenuated slightly by adjusting for the need for an interpreter, and reduced further by adding the predictor of a psychotic illness, to the point that the odds ratios for involuntary admission for all ethnicities were no longer significantly different. The addition of the psychosis predictor also markedly improved the goodness of fit of the model. To further investigate the importance of psychosis in moderating the association between ethnicity and involuntary admission, a *post hoc* analysis was performed with an interaction term included in the model. This showed that in those with a diagnosis of psychosis, ethnicity did not significant influence the likelihood of involuntary admission. In those without a diagnosis of psychosis, being of Black, Asian, Mixed or other ethnicity was associated with an increased likelihood of involuntary admission (odds ratio 3.3, 95% CI 1.1–10.0). None of the models achieved an excellent goodness of fit (typically defined as McFadden’s *R* ^2^ ≥ 0.2), suggesting other, unmeasured, variables are also likely to be important.

**Table 2:** Logistic regression testing predictors of involuntary admission in women admitted for the first time to the Mother and Baby Unit (2021–2024), Livingston, Scotland Table 2 long description.

Ethnicity	White British	White not British	Black, Asian, Mixed or other ethnicity	Goodness of fit of model (McFadden’s *R* ^2^)
Simple model: ethnicity alone				
Odds ratio (95% CI)	1	2.1 (1.0–4.3)^*^	2.7 (1.4–5.2)**	0.024
*z*-statistic		1.96	3.041	
*P*-value		0.04997	0.00236	
Adjusted model: ethnicity plus need for an interpreter				
Adjusted odds ratio (95% CI)	1	1.7 (0.8–3.7)	2.2 (1.1–4.5)^*^	0.029
*z*-statistic		1.27	2.22	
*P*-value		0.21	0.0264	
Adjusted model: ethnicity plus need for an interpreter plus diagnosis of psychosis				
Adjusted odds ratio (95% CI)	1	1.6 (0.7–3.8)	1.9 (0.8–4.3)	0.178
*z*-statistic		1.03	1.67	
*P*-value		0.30	0.09	
Adjusted model assessing psychosis as moderator of effect of ethnicity: ethnicity plus need for an interpreter plus diagnosis of psychosis plus ethnicity × diagnosis of psychosis				
No diagnosis of psychosis				
Adjusted odds ratio (95% CI)	1	2.9 (0.9–9.7)	3.3 (1.1–10.0)^*^	0.183
*z*-statistic		1.73	2.14	
*P*-value		0.08	0.0324	
Diagnosis of psychosis				
Adjusted odds ratio (95% CI)	1	1.0 (0.3–2.9)	1.3 (0.5–3.4)	
*z*-statistic		−0.05	0.50	
*P*-value		0.96	0.62	

* *P* < 0.05, **P < 0.01.

## Discussion

The key finding of this service evaluation was that in 12 years of admissions from a Scottish MBU, patients from an ethnic minority background were admitted involuntarily twice as often as White British patients. Higher frequency of psychotic illness contributed to this finding, but an effect of ethnicity was also seen in those without a diagnosis of psychosis.

Our findings are consistent with work showing increased involuntary admissions in people of ethnic minority background in MBUs outside of Scotland^4^ and outside of perinatal settings.^10^ The range of potential explanations for our findings are wide,^11^ including (but not limited to) institutional and/or interpersonal racism (deliberate or unconscious), differences in patient pathways to services, differences in frequencies of diagnoses, language barriers and cultural barriers. This service evaluation was able to address two possible explanations: psychosis and language barrier as shown by the need for an interpreter.

Psychosis was found to be a predictor of involuntary admission in this setting, consistent with findings from meta-analysis of predictors of involuntary admission internationally.^12^ We also found that a higher proportion of patients from an ethnic minority were admitted with a psychotic illness. In a *post hoc* analysis, which included an interaction between ethnicity and psychotic illness, we found no evidence that ethnicity was associated with involuntary admission among patients with a psychotic illness. However, among patients without a diagnosis of psychosis, being of Black, Asian, Mixed or other ethnicity was associated with an increased likelihood of involuntary admission. This may reflect the strong association of psychosis with involuntary admission, with the presence of a psychotic illness potentially overriding other factors, which had an impact only in the absence of psychosis.

Why might a greater proportion of patients who are not White British and are admitted to an MBU have a psychotic illness than their White British peers? Potentially many steps on the patient journey could be relevant. It may be because patients who are from minority ethnicities are less likely to be assessed by secondary care with other illnesses that could result in admission; for example, remaining at home with undetected severe depression. It may be because patients present for treatment later, meaning that symptoms are more severe at presentation; for example, severe depression can worsen to become psychotic depression. A systematic review of the lived experience of women from ethnic minority backgrounds who had experienced perinatal mental illness in the UK found themes including that some women were not aware of the existence of perinatal mental disorders, felt that mental illness was culturally unacceptable, coped with their symptoms by minimising them and were not aware of support services or avoided accessing them.^13^

Based on work in other contexts, it is also plausible that there is a true higher frequency of psychotic illness in mothers and birthing people living in Scotland who are of minority ethnicity. It is well-established that psychosis has a higher incidence in migrants to high-income countries and their children, particularly people of Black ethnicity.^14^ This effect is likely driven by social marginalisation and language differences rather than intrinsic to that ethnicity.^15^ A higher frequency of psychotic illness could also partly explain the higher proportion of admissions for patients of minority ethnicity than expected from extrapolation of data on ethnicity recorded by the census and Scotland’s maternity registry.

Another possible explanation for the association between ethnicity and involuntary admission is clinician perception of ‘dangerousness’ being influenced by a patient’s ethnicity.^16^ A recent Mental Welfare Commission report in Scotland found that detained people who were of Black or Mixed ethnicity were perceived as a greater risk to themselves or others compared with White Scottish people, who were more likely to be considered only a risk to themselves, particularly in the case of women (48% of Black or Mixed ethnicity women viewed as presenting a risk to others versus 34% of White Scottish women).^17^

This report also conducted qualitative interviews with people with lived experience of seeking mental health support in Scotland, finding that for some, language was a significant hurdle in accessing and using services.^17^ This was also found by a systematic review of lived experience with women who accessed perinatal services, which found that language and culture was a barrier, as was perceived negative attitudes from professionals.^13^ We did not find a significant effect of the need for an interpreter independent of ethnicity, but we could not account for more subtle challenges in communication and expectation below the threshold of needing an interpreter.

We also found an 11% missingness rate for coding ethnicity in electronic records. The reasons for this are unclear – it may reflect staff not asking about ethnicity, or it may reflect staff asking, but not inputting this data electronically. Either way, this is an area that could be improved on, as without accurate data, the relationship between ethnicity and healthcare parameters cannot be determined. Identification of ethnicity in healthcare records has been identified as one way for systems to tackle racism in maternal health.^11^

### Strengths and limitations

This is an observational study and as such can find evidence of association, but not causation. We classed people of many different ethnicities together, and so may have missed effects specific to particular ethnicities. Relying on routinely collected electronic records meant that we were not able to measure all the known predictors of involuntary admission, such as socioeconomic status and previous use of the Mental Health Act. The findings may not be generalisable to other settings, as our sample covers a relatively long time period, mental health acts vary between countries and the risks in the perinatal period are different to other times of life (with risk to the foetus/infant always considered). The major strength of this study is that it represents a complete description of a service since its inception.

### Future work

Future work is needed to understand why patients from ethnic minority backgrounds admitted to MBUs in Scotland and England are more likely to be admitted involuntarily. It will be important to ask patients themselves, and staff members. The impact of ethnicity and race on access to health services in general is an area of increasing focus, with NHS Lothian recently committing to being anti-racist.^18^

The possibility that patients from ethnic minority backgrounds are more likely to be admitted to a MBU and to experience psychosis in the perinatal period should also be better quantified with population-level data and additional covariates. If replicated, interventions to reduce psychosis, such as early intervention teams and culturally informed care, may go some way to reducing these differences.

The key implications of this work for clinicians are to consider how a patient’s experience of mental healthcare is influenced by their ethnicity, ensure that data on ethnicity is collected and to take steps to reduce ethnic disparities in mental healthcare wherever opportunities are identified.

## Data Availability

The data that support the findings of this study are not available as they contain information that could compromise the privacy of patients.

## About the authors

**Katie F. M. Marwick,** MRCPsych, PhD, is a Senior Clinical Research Fellow at the Division of Psychiatry, University of Edinburgh, Edinburgh, UK; and an Honorary Consultant Psychiatrist with the Perinatal Mental Health Service, NHS Lothian, Edinburgh, UK. **Zia J. Low,** BSc, MBBS, is a Foundation Year 4 doctor in the Acute Medicine Unit Raigmore Hospital, Inverness, UK. **Kelly Fleetwood**, MSc, is a medical statistician at the Usher Institute, University of Edinburgh, Edinburgh, UK.

## Table 1 Long description

The table presents demographic and clinical characteristics of women admitted to the Mother and Baby Unit, categorized by ethnicity. It has 10 rows and 5 columns. The columns are labeled Ethnicity, White British, White not British, and Black, Asian, Mixed or other ethnicity. The rows include n (% of total), Age in years, n (% within group), Admission length in days, mean (s.d.), Interpreter required, n (% within group), Diagnosis, n (% within group), and Admitted involuntarily, n (%). Row 1: Ethnicity, White British, White not British, Black, Asian, Mixed or other ethnicity. Row 2: n (% of total), 364 (80.9), 38 (8.4), 48 (10.7). Row 3: Age in years, n (% within group), <=24, 103 (28.3), 7 (18.4), 9 (18.8). Row 4: Age in years, n (% within group), 25-29, 94 (25.8), 9 (23.7), 18 (37.5). Row 5: Age in years, n (% within group), 30-34, 85 (23.4), 12 (31.6), 16 (33.3). Row 6: Age in years, n (% within group), >=35, 82 (22.5), 10 (26.3), 5 (10.4). Row 7: Admission length in days, mean (s.d.), 29.4 (33.8), 32.2 (23.0), 29.0 (34.7). Row 8: Interpreter required, n (% within group), 0, 9 (23.7), 11 (22.9). Row 9: Diagnosis, n (% within group), Psychotic disorder, 109 (29.9), 17 (44.7), 24 (50.0). Row 10: Diagnosis, n (% within group), Mood disorder, 99 (27.2), 11 (28.9), 9 (18.8). Row 11: Diagnosis, n (% within group), Anxiety disorder, 62 (17.0), 5 (13.2), 8 (16.7). Row 12: Diagnosis, n (% within group), Personality disorder, other or no disorder, 94 (25.8), 5 (13.2), 7 (14.6). Row 13: Admitted involuntarily, n (%), 66 (18.1), 12 (31.6), 18 (37.5).

Navigate back to Table 1.

## Table 2 Long description

The table presents data on the predictors of involuntary admission for women admitted to the Mother and Baby Unit in Livingston, Scotland, from 2021 to 2024. It includes odds ratios, z-statistics, and p-values for different ethnic groups under various models. The table has four main sections: a simple model with ethnicity alone, an adjusted model with ethnicity plus need for an interpreter, an adjusted model with ethnicity plus need for an interpreter plus diagnosis of psychosis, and an adjusted model assessing psychosis as a moderator of the effect of ethnicity. Each section provides data for White British, White not British, and Black, Asian, Mixed or other ethnicity groups. The table also includes a column for the goodness of fit of the model (McFadden's R²).

Navigate back to Table 2.
